# Review of Network Meta-Analyses on the Efficacy of Chemopreventive Agents on Colorectal Adenomas and Cancer

**DOI:** 10.1177/10732748251344481

**Published:** 2025-05-20

**Authors:** Yibing Ruan, Chantelle Carbonell, Karen Brown, Robert J. Hilsden, Darren R. Brenner

**Affiliations:** 1 Department of Oncology, Cumming School of Medicine, University of Calgary, Calgary, AB, Canada; 2 Department of Cancer Epidemiology and Prevention Research, Cancer Care Alberta, Alberta Health Services, Calgary, AB, Canada; 3 Leicester Cancer Research Centre, University of Leicester, Leicester, UK; 4 Department of Medicine, Cumming School of Medicine, University of Calgary, Calgary, AB, Canada; 5 Department of Community Health Sciences, Cumming School of Medicine, University of Calgary, Calgary, AB, Canada

**Keywords:** colorectal cancer, colorectal adenoma, chemoprevention, preventive therapy, therapeutic prevention, chemopreventive agents

## Abstract

**Background:**

Colorectal cancer (CRC) is the third most diagnosed cancer and the second leading cause of cancer-related death worldwide. Colorectal adenomas (CRAs) are a crucial precursor for CRC and a target for preventive strategies. Recent network meta-analyses (NMAs) of randomized controlled trials (RCTs) suggest that chemopreventive agents (CPAs) are associated with reductions in CRC incidence. However, the quality of this evidence is low due to significant variability in the methods and types of studies assessed.

**Purpose:**

Our study reviewed the efficacy and safety of CPAs on CRAs or CRCs evaluated in NMAs of RCTs and assessed the quality of all published NMAs on CPAs.

**Research Design:**

We searched PubMed, Embase, and Cochrane Library for studies published from inception to July 29, 2024. We included all NMAs assessing the efficacy and safety of CPAs on CRC in both average-risk (general population) and high-risk (previous history of adenoma/CRC) populations.

**Results:**

Nine NMAs comparing 15 different interventions were included. Aspirin and non-aspirin non-steroidal anti-inflammatory drugs (NSAIDs) such as celecoxib were the most studied. Aspirin demonstrated efficacy against the development of any CRA and low-dose aspirin was consistently more protective than high-dose aspirin. However, the effect of aspirin against advanced CRA was not statistically significant. Concerns for long-term aspirin use included an increased risk of gastrointestinal bleeding and ulceration, but when evaluating all serious adverse events (SAEs), aspirin users did not have an increased risk compared to controls. Non-aspirin NSAIDs showed better efficacy against advanced CRA. However, the use of non-aspirin NSAIDs such as celecoxib was associated with significantly increased risk of SAEs, particularly cardiovascular disease events.

**Conclusions:**

Considering the balance of efficacy and safety, low-dose aspirin is currently the best option for chemoprevention of CRA/CRC. Future research is needed to better characterize the patient subgroups that benefit most and to develop new, more effective CPAs.

## Introduction

Colorectal cancer (CRC) is the third most commonly diagnosed cancer and the second leading cause of cancer-related death worldwide.^1^ Approximately 1.9 million new diagnoses of CRC and roughly 900 000 CRC-related deaths were reported worldwide in 2022.^1^ Organized screening programs have led to substantial reductions in CRC incidence rates in recent years through the detection and removal of precancerous polyps, such as colorectal adenomas (CRAs).^1-4^ CRAs are a crucial precursor for CRC and a target for preventive strategies.^3^ Chemoprevention, a term that is increasingly preferred as “preventive therapy”, or “therapeutic prevention”, refers to the use of certain agents or substances to reduce a person’s risk of developing a disease.^5^ Recent advances in CRC chemoprevention have focused on leveraging repurposed pharmacological agents and dietary interventions to reduce the burden of CRC beyond screening.^6^ A published umbrella review of existing systematic reviews and meta-analyses on chemoprevention for CRC suggest that CRC chemopreventive agents (CPAs), such as aspirin, non-steroidal anti-inflammatory drugs (NSAIDs), magnesium, and folate are associated with reductions in CRC incidence.^6^ However, the quality of this evidence is low as the studies assessed demonstrated substantial heterogeneity in their methods and study types.^6^ Furthermore, traditional meta-analysis only makes direct comparison of two interventions. Indirect comparisons and ranking of multiple interventions are not feasible with pairwise meta-analysis.

Network meta-analysis (NMA) of randomized controlled trials (RCTs) as an approach to synthesizing evidence is highly valuable when multiple interventions are compared. Compared to a traditional meta-analysis, NMA allows multiple intervention comparisons even when two interventions have not been directly compared in any RCTs.^7^ However, for these indirect comparisons to be valid, two key assumptions must be held. First, the effect modifiers of the relative intervention effects, for example, patient characteristics, must be similar across trials of different interventions. Second, the intervention effect estimates for a particular pairwise comparison must be consistent between the direct and indirect comparisons.^7,8^ Otherwise, NMA are like traditional meta-analyses in methods, can be performed with fixed- or random-effects models, and within a frequentist or Bayesian framework. In this study, we aimed to review the efficacy and safety of all CPAs on CRAs or CRCs that have been evaluated in NMAs of RCTs. We also aimed to assess the quality of all published NMAs on CPAs.

## Methods

### Search Strategy

We identified relevant studies through a systematic search of PubMed, Embase, and Cochrane Library databases using the search strategy as illustrated in Table S1. The search was restricted to studies published from inception to July 29, 2024.

### Study Selection and Patient Population

Our selected studies included all NMAs assessing the efficacy and safety of CPAs on CRAs or CRCs in both average-risk (general population) and high-risk (previous history of adenoma/CRC) populations. The following inclusion criteria were applied in selecting studies: (1) Published studies of network meta-analysis on chemopreventive agents; (2) Study population was either ‘high-risk’ patients with previous history of CRC or advanced CRA (adenoma ≥10 mm, with tubulovillous or villous histology, or with high-grade dysplasia), regardless of whether they have genetic predisposition of CRC, or ‘average-risk’ individuals with no history of CRC or advanced CRA; (3) Outcomes were the risk ratios (RRs) of the detection rate of CRC, advanced CRAs, or any CRAs, and of the adverse event (AE) rate. (Table S2)

## Results

**Figure 1.**
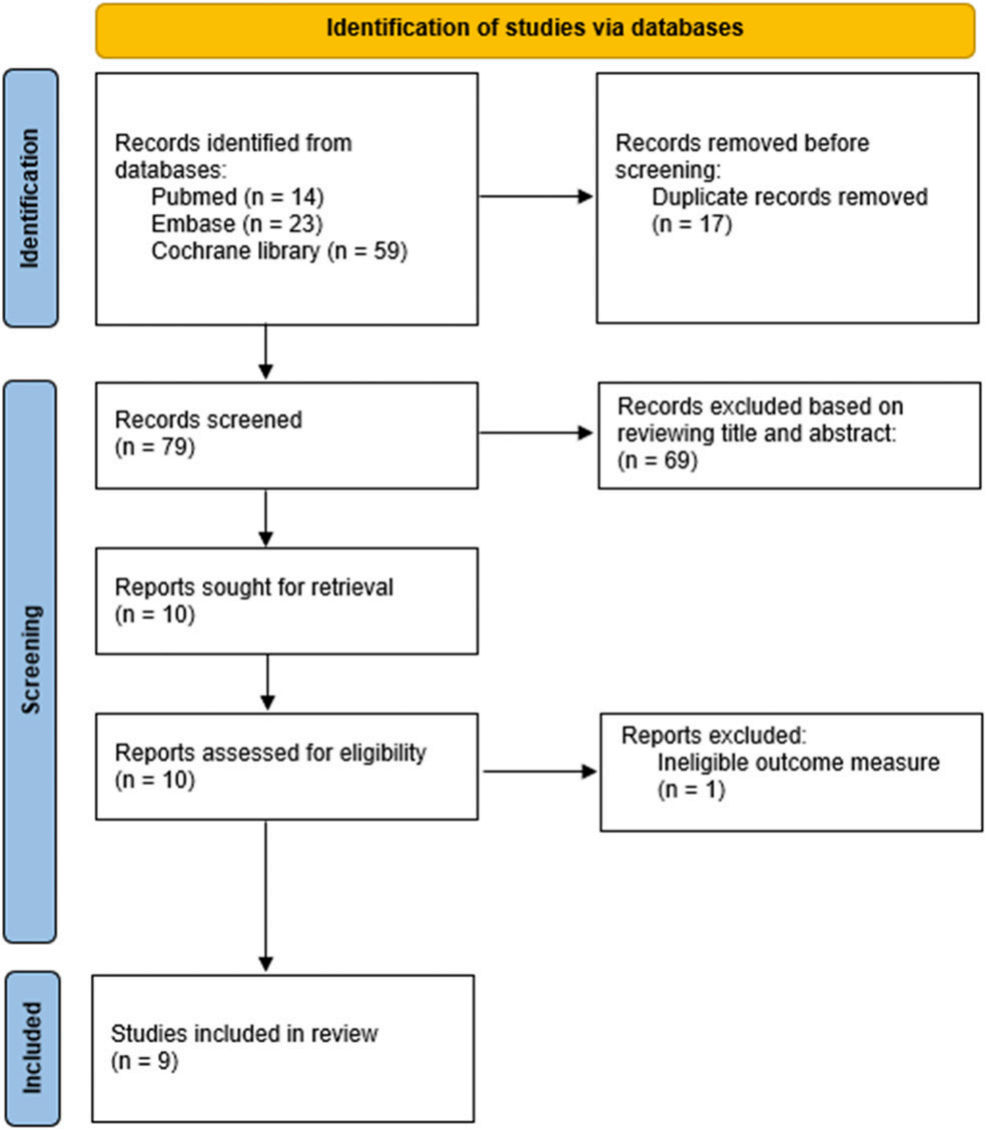
PRISMA Diagram.

The initial search resulted in 79 studies eligible for abstract screening. 69 studies did not pass screening because they were either not NMA or NMA on topics not related to chemoprevention. Among the ten NMAs that were relevant to chemoprevention of recurrent CRA/CRC, one was excluded because it included observational studies, and the outcome included tumor markers instead of endoscopically confirmed adenomas or cancer,^9^ resulting in nine NMAs included in the final analysis (Figure 1). An overview of the included NMAs is summarized in Table 1. All NMAs were published between 2016 and 2024, including eligible RCTs from 1999 to 2022. As there is currently no tool to evaluate the quality of NMA, we included three metrics to indicate whether the NMA has used GRADE or ROB to assess the RCTs, has performed test of consistency, and has conducted sensitivity analysis (Table 1). Most NMAs focused on recurrent CRAs among high-risk individuals with previous CRAs or CRCs, while two NMAs also studied the chemoprevention effect on the general population with average risk.^10,11^ One NMA included both high-risk and average risk population, as well as subjects with Lynch syndrome or familial adenomatous polyposis.^12^ A total of 25 interventions (with different doses of aspirin and combination of CPAs as individual interventions) were assessed among all NMAs. We focused on 15 interventions that were included in at least two NMAs (Table 2).

**Table 1.** Summary of the Included Network Meta-Analysis (NMAs).

First Author	Country /origin of institute	Title	Publication Year	Study year span	No. RCT included	No. Interventions	No. Pts	Statistical framework	Population characteristics	Outcome	GRADE/ROB	Evaluation of consistency	Sensitivity analysis
Dulai, P.	USA	Chemoprevention of colorectal cancer in individuals with previous colorectal neoplasia: Systematic review and network meta-analysis	2016	1999-2015	14	9	12 234	Bayesian	High risk	Adv. Neoplasia	Y	Y	Y
Veettil, S.	Malaysia	Effects of chemopreventive agents on the incidence of recurrent colorectal adenomas: a Systematic review with network meta-analysis of randomized controlled trials	2017	1988-2016	20	12	12 625	Frequentist	High risk	Any adenoma; Adv. Adenoma	Y	Y	Y
Veettil, S.	Malaysia	Efficacy and safety of chemopreventive agents on colorectal cancer incidence and mortality: Systematic review and network meta-analysis	2018	1988-2013	21	12	281 063	Frequentist	General population	CRC (0-10y); CRC (10-20y)	Y	Y	Y
Veettil, S.	Malaysia	Very low dose aspirin and surveillance colonoscopy is cost effective in secondary Prevention of colorectal cancer in individuals with advanced adenomas: Network metaanalysis and cost effectiveness analysis	2021	1999-2018	14	8	NR	Frequentist	High risk	Adv. Adenoma	Y	Y	Y
Heer, E.	Canada	The efficacy of chemopreventive agents on the incidence of colorectal adenomas: A systematic review and network meta-analysis	2022	1988-2019	33	12	20 925	Bayesian	High risk	Any adenoma; Adv. Adenoma	Y	Y	Y
Shah, D.	Australia	Aspirin chemoprevention in colorectal cancer: network meta-analysis of low, moderate, and high doses	2023	1998-2018	11	3	92 550	Bayesian	High risk	CRC	Y	N	N
Ma, Y	China	Chemoprevention of colorectal cancer in general population and high-risk population: a Systematic review and network meta-analysis	2023	1993-2021	32	13	278 694	Frequentist	General population; High risk	Any adenoma; Adv. Adenoma; CRC	Y	Y	Y
Hoang, K	Taiwan	Oral aspirin for preventing colorectal adenoma recurrence: A systematic review and network meta-analysis of randomized controlled trials	2024	2003-2021	8	2	3011	Frequentist	High risk	Any adenoma	Y	N	Y
Tan	China	Preventive effects of chemical drugs on recurrence of colorectal adenomas: Systematic review and Bayesian network meta-analysis	2024	2000-2022	45	12	35 590	Bayesian	High risk	Any adenoma	Y	Y	N

**Table 2.** Efficacy of Chemopreventive Agents (CPAs).

Outcome	First Author	Publication Year	Population characteristics	NSAID (non-ASA)	DFMO + Sulindac	VLD-ASA	LD-ASA	HD-ASA	ASA, any dose	Calcium	Vitamin D	Calcium + Vitamin D	ASA + Calcium + Vitamin D	Folate/folic acid	ASA + Folate	Antioxidant	Berberine	UDCA
Any adenoma	Veettil, S.	2017	High risk	CEL400: 0.70 (0.64, 0.76)			0.83 (0.70, 0.99)	0.93 (0.67, 1.29)		0.83 (0.75, 0.93)	0.98 (0.84, 1.13)	0.86 (0.66, 1.12)	0.94 (0.60, 1.45)	1.06 (0.87, 1.28)	0.91 (0.70, 1.19)	0.77 (0.54, 1.11)		
				CEL800: 0.62 (0.55, 0.69)														
	Heer, E.	2022	High risk	CEL: 0.71 (0.48, 1.04)	0.24 (0.10, 0.55)		0.69 (0.52, 0.91)	0.85 (0.69, 1.05)	0.77 (0.59, 1.00)	0.78 (0.56, 1.08)	0.91 (0.58, 1.39)	0.87 (0.56, 1.33)	0.94 (0.54, 1.64)	1.08 (0.79, 1.49)	HD: 0.82 (0.51, 1.33)	0.84 (0.63, 1.07)	0.72 (0.37, 1.40)	0.90 (0.46, 1.74)
	Ma, Y	2023	Avg risk	0.59 (0.44, 0.79)			0.69 (0.42, 1.13)	0.88 (1.64, 1.20)		0.88 (0.55, 1.41)	1.02 (0.70, 1.50)	0.93 (0.50, 1.74)	0.91 (0.43, 1.94)	0.90 (0.66, 1.22)	0.76 (0.40, 1.44)			0.89 (0.46, 1.71)
	Ma, Y	2023	High risk	0.58 (0.43, 0.79)			0.70 (0.45, 1.11)	0.91 (0.66, 1.27)		0.92 (0.59, 1.44)	1.21 (0.65, 2.23)	1.00 (0.54, 1.84)	0.91 (0.46, 1.83)	1.13 (0.78, 1.63)	0.83 (0.46, 1.51)			0.89 (0.49, 1.59)
	Hoang, K	2024	High risk				0.70 (0.54, 0.91)	0.92 (0.77, 1.10)										
	Tan	2024	High risk	0.84 (0.76, 0.92)	0.34 (0.25, 0.44)				0.90 (0.84, 0.96)	0.94 (0.90, 1.01)	1.02 (0.97, 1.09)	0.98 (0.92, 1.05)		0.89 (0.79, 0.98)		0.98 (0.94, 1.01)	0.79 (0.68, 0.92)	
Adv. Adenoma	Veettil, S.	2017	High risk	CEL400: 0.48 (0.38, 0.60)			0.84 (0.46, 1.55)	0.88 (0.61, 1.25)		1.01 (0.74, 1.38)	1.19 (0.78, 1.82)	0.95 (0.66, 1.37)		1.21 (0.82, 1.78)	LD: 0.54 (0.28, 1.02)	0.45 (0.16, 1.23)		
				CEL800: 0.37 (0.26, 0.51)											HD: 0.81 (0.55, 1.19)			
	Veettil, S.	2021	High risk	CEL400: 0.45 (0.35, 0.58)		0.49 (0.31, 0.78)	0.79 (0.63, 1.00)			1.00 (0.74, 1.35)	1.17 (0.79, 1.73)	0.92 (0.64,1.33)	0.75 (0.24, 2.31)					
				CEL800: 0.36 (0.25, 0.52)														
	Heer, E.	2022	High risk	CEL: 0.51 (0.25, 0.88)	0.06 (0.00, 0.40)		0.57 (0.30, 1.06)	0.82 (0.56, 1.18)	0.73 (0.37, 1.25)	1.24 (0.64, 2.45)	1.20 (0.61, 2.33)	1.03 (0.52, 2.02)	0.76 (0.20, 2.72)	1.25 (0.81, 1.90)	HD: 0.64 (0.32, 1.24)	1.09 (0.49, 2.27)	0.57 (0.20, 1.48)	0.80 (0.36, 1.66)
	Ma, Y	2023	Avg risk	0.63 (0.43, 0.92)			0.72 (0.42, 1.24)	0.76 (0.51, 1.13)		0.94 (0.57, 1.54)	1.04 (0.51, 2.12)	0.87 (0.42, 1.79)	0.74 (0.21, 2.63)	1.06 (0.75, 1.49)	0.58 (0.29, 1.17)			0.82 (0.45, 1.48)
Adv. Neoplasia	Dulai, P.	2016	High risk	0.37 (0.24-0.53)			0.71 (0.41, 1.23)	1.12 (0.59, 2.10)		1.00 (0.66, 1.52)	1.19 (0.65, 2.15)	0.91 (0.52, 1.63)	0.71 (0.18, 2.49)	1.32 (0.85, 2.00)	0.73 (0.43, 1.19)			
CRC (0-10y)	Veettil, S.	2018	Avg risk			0.89 (0.63, 1.26)	1.15 (0.75, 1.74)	0.91 (0.55, 1.53)		0.19 (0.01, 3.60)	1.03 (0.59, 1.82)	1.06 (0.78,1.44)		1.00 (0.14, 7.14)		0.94 (0.81, 1.10)		
CRC (10-20y)	Veettil, S.	2018	Avg risk			0.81 (0.67, 0.98)	1.03 (0.83, 1.27)	0.74 (0.57, 0.97)				0.96 [0.81, 1.13]				1.07 (0.89,1.28)		
CRC	Shah, D.	2023	Mixed risk				0.95 (0.78, 1.16)	0.73 (0.25, 2.08)										
CRC	Ma, Y	2023	Avg risk	0.68 (0.28, 1.62)			1.21 (0.83, 1.75)	1.03 (0.58, 1.83)			1.09 (0.56, 2.12)	1.09 (0.61, 1.94)		1.34 (0.78, 2.32)				

Footnote: Antioxidants include any interventions with vitamin A, B6, B12, C, and E, α-tocopherol, beta-carotene, selenium, green tea extracts, either as single agent or as combinations.

Abbreviations: Adv, advanced; ASA, Acetylsalicylic acid (aspirin); Avg, average; CPA, chemopreventive agent; CRC, colorectal cancer; DFMO, difluoromethylornithine; HD, high-dose; LD, low-dose; NSAID, non-steroidal anti-inflammatory drug; UDCA, ursodeoxycholic acid; VLD, very low-dose.

Aspirin was the most studied agent for preventing CRAs. All nine NMAs included aspirin in their analyses. Most NMAs presented odds ratios (ORs) for aspirin by low (80 – 160 mg/day) or high (160 – 325 mg/day) doses. For the incidence of any CRA, the protective effect of low-dose aspirin was consistently better than that of high-dose aspirin.^11,13-15^ However, in terms of advanced adenoma, all NMAs suggested that effect estimates were not statistically significant with either low- or high-dose aspirin intervention. Interestingly, one NMA showed that very low dose of aspirin (<100 mg/day) reduces the risk of advanced adenoma by 51% (RR: 0.49, 95% CI: 0.31-0.78).^16^ Three NMAs also investigated the effect of aspirin on preventing CRC. Aspirin did not reduce the risk of CRC among a mixed population of average risk, high risk, and individuals with genetic predisposition^12^ or among the average-risk population with less than 10 years of follow-up.^10,11^ Among the general population with longer than 10 years of follow-up, both very low dose and high-dose aspirin demonstrated a significant protective effect.^10^ The major safety concern of long-term aspirin use was gastrointestinal (GI) bleeding and ulceration. A significantly increased risk was observed among both the high-risk individuals^12^ or the general population^10^ in a dose-dependent manner. On the other hand, when looking at all serious AEs, including GI bleeding, cardiovascular or non-cardiovascular complications, hospitalization, and death, aspirin users did not have increased risk compared to the control group.^11,16,17^

Non-aspirin NSAIDs, such as cyclooxygenase-2 (Cox-2) inhibitors and sulindac, are an important family of agents for preventing CRAs. Six NMAs included non-aspirin NSAIDs, all of which were on high-risk individuals with a history of adenoma or CRC^11,13,14,16-18^ and one NMA also provided effect estimates on the general population.^11^ Non-aspirin NSAIDs were consistently ranked among the most effective agents in preventing recurrent advanced adenoma.^11,13,14,16,17^ In terms of any CRA, the RRs of non-aspirin NSAIDs were less consistent, ranging from 0.59^11^ to 0.84.^18^ Two NMAs observed the strongest prevention effect with a combination of difluoromethylornithine and sulindac.^14,18^ However, it was based on one RCT with moderate to high risk of bias.^19^ The safety of non-aspirin NSAIDs was a concern for long-term use. An estimated 20-30% of increased risk of serious AEs was found in three NMAs.^11,16,17^ The risk of cardiovascular and thrombotic AEs with long-term use of non-aspirin NSAIDs is particularly elevated in patients with pre-existing atherosclerotic heart diseases.^20^

Observational studies have observed a link between low levels of dietary calcium and an increased risk of CRC,^21^ and between higher vitamin D levels and a reduced risk of CRC.^22^ Therefore, it was suggested that increasing calcium intake and/or vitamin D supplementation may lower the risk for recurrent CRA. However, except for one NMA which reported a 17% decreased risk of any CRA (RR: 0.83, 95% CI: 0.75-0.93),^13^ no protective effects of calcium and vitamin D supplementation, alone or in combination, were observed among the high-risk individuals.^11,13,14,16-18^ Calcium and vitamin D had no effects on CRA or CRC either among the general population.^10,11^ Although calcium ranked the top agent in reducing early risk of CRC in one NMA,^10^ the confidence interval (CI) was extremely wide, indicating very high uncertainty (RR: 0.19, 95% CI: 0.01-3.60). In addition, five NMAs reported the combination of calcium, vitamin D, and aspirin among high-risk individuals.^11,13,14,16,17^ The effect estimates were similar to those of aspirin alone. It should be noted that although individual RCTs may account for baseline level of calcium or vitamin D among the participants, it was not possible to adjust for this variable in NMAs. There might be a protective effect among individuals with deficiency of calcium or vitamin D. Surprisingly, the safety of calcium supplementation among high-risk individuals seemed to be questionable. Among four NMAs reporting the AEs for calcium, two reported 38% and 22% increased risk of serious AEs, including death, hospital admission, severe gastrointestinal bleeding, vascular complications, or discontinuation of treatment due to serious or severe events, which were statistically significant.^16,17^ However, no increased risk was observed for vitamin D or the combination of calcium and vitamin D.^11,16-18^

Folate and folic acid have been controversial in the association with CRC. While observational studies suggested that higher folate intake was associated with reduced risk of CRC, RCTs did not find a beneficial role in CRC and even a possible increase in CRA.^23^ Folate also potentially interferes with chemotherapy among CRC patients undergoing active therapy.^24^ Chemotherapeutical drug fluoropyrimidines, such as 5-fluorouracil (5-FU) or capecitabine, primarily target thymidylate synthase (TS), a key enzyme in nucleotide biosynthesis and folate metabolism. Therefore, intermediate folate derivatives may form a stable complex with TS and 5-FU and influence the TS inhibition rate. Observational studies have shown that higher serum level of folate during chemotherapy was associated with a higher risk of toxicity.^24^ The lack of preventive effect of folate in CRC or CRA was observed in most NMAs,^10,11,13,14,17^ except for the latest NMA, which reported a 11% reduction in any CRA (RR: 0.89, 95% CI: 0.79-0.98).^18^ The reason for this discrepancy is unclear. It is possible that the included RCTs for folate are different in this NMA, as the authors also reported a significantly higher risk of AEs (RR: 1.21, 95% CI: 1.08-1.35) in contrast to other NMAs (Table 3).

**Table 3.** Safety of Chemopreventive Agents (CPAs).

First Author	Publication Year	Outcome	NSAID (non-ASA)	DFMO + Sulindac	VLD-ASA	LD-ASA	HD-ASA	ASA, any dose	Calcium	Vitamin D	Calcium + Vitamin D	ASA + Calcium + Vitamin D	Folate/folic acid	ASA + Folate	Antioxidant	Berberine	UDCA
Dulai, P.	2016	Serious adverse events*	1.23 (0.95, 1.64)			0.78 (0.43, 1.38)	1.06 (0.76, 1.49)		1.38 (1.07, 1.89)	1.10 (0.74, 1.70)	1.11 (0.76, 1.70)	0.80 (0.54, 1.51)	0.85 (0.59, 1.22)	1.21 (0.83, 1.77)			
Veettil, S.	2018	Major GI bleeding			1.44 (1.15, 1.81)	1.85 (1.22, 2.81)	4.04 (1.86, 8.76)										
Veettil, S.	2021	Serious adverse events*	CEL400: 1.16 (0.97,1.38) CEL800: 1.27 (1.00,1.62)		0.82 (0.47,1.41)	0.97 (0.78,1.21)			1.22 (1.02,1.44)	1.05 (0.82,1.35)	1.07 (0.85,1.34)	0.92 (0.64, 1.32)					
Shah, D.	2023	GI bleeding and ulceration				1.32 (1.09, 1.79)	1.64 (1.05, 2.56)										
Ma, Y	2023	Serious adverse events**	1.29 (1.13, 1.47)					0.95 (0.76, 1.19)	0.87 (0.57, 1.31)	1.06 (0.91, 1.24)		0.95 (0.57, 1.60)	1.10 (0.77, 1.56)	1.03 (0.56, 1.91)			
Tan	2024	Adverse events***		3.19 (1.80, 6.45)					1.11 (0.92, 1.32)	0.98 (0.85, 1.12)			1.21 (1.08, 1.35)		1.13 (1.02, 1.23)	0.94 (0.43, 2.05)	1.01 (0.63, 1.45)

*Defined as events resulting in death, admission to hospital related to an adverse event, severe gastrointestinal bleeding, cardiovascular (CV) or non-CV complications, or discontinuation of treatment because of an adverse event or events that were graded as serious or severe by original study authors.

**Defined as events resulting in death, hospital admission related to adverse events, cardiovascular events, stroke, and gastrointestinal bleeding.

***Undefined.

Abbreviations: ASA, Acetylsalicylic acid (aspirin); CPA, chemopreventive agent; DFMO, difluoromethylornithine; HD, high-dose; LD, low-dose; NSAID, non-steroidal anti-inflammatory drug; UDCA, ursodeoxycholic acid; VLD, very low-dose.

Other less commonly studied agents include antioxidants, berberine, and ursodeoxycholic acid (UDCA) (Table 2). In NMAs, antioxidants are a category of interventions that include vitamin A, B6, B12, C, and E, α-tocopherol, beta-carotene, selenium and green tea extracts, either as single agent or as combinations. Four NMAs examined the effect of antioxidants, and no association was found for CRA or CRC.^10,13,14,18^ No association was found for UDCA either.^11,14^ Two NMAs examined berberine, a traditional Chinese medicine suggested to have anti-inflammatory and antitumor effects. One NMA found a non-significant effect of preventing any adenoma based on one RCT (RR: 0.72, 95% CI: 0.37-1.40).^14^ The other NMA found a significant effect based on three RCTs, two of which were published in Chinese language (RR: 0.79, 95% CI: 0.68-0.92).^18^

## Discussion

Among all CPAs we have reviewed, Aspirin and non-aspirin NSAIDs such as celecoxib were the most well-studied. Aspirin demonstrated efficacy against the development of any CRA, but the effect against advanced CRA was not statistically significant. Non-aspirin NSAIDs have better efficacy against advanced CRA. However, the use of non-aspirin NSAIDs such as celecoxib was associated with significantly increased risk of serious AEs, particularly cardiovascular disease (CVD) events. Therefore, non-aspirin NSAIDs for CRC prevention are currently approved for use in people with familial adenomatous polyposis (FAP), and could be considered for people without underlying risk of CVD.^25^ On the other hand, low-dose aspirin is a more practical option for the prevention of recurrent CPA among both the high-risk and the general population, which only increases the risk of GI bleeding but not other serious AEs. In 2016, the US Preventive Services Task Force (USPSTF) endorsed low-dose aspirin for CRC prevention among adults aged 50-59 without an increased risk of bleeding.^26^ However, USPSTF withdrew this recommendation in 2022 because of updated RCT results indicating no reduced risk in CRC prevention.^27^ Although this decision was criticized by some researchers for biasedly selecting and interpreting evidence,^28^ it highlighted the urgency to better understand the ideal prevention windows, duration, and the characteristics of the patient subgroups who benefit more from aspirin use, and those individuals who are at greater risk of side effects.

It is important to note that all NMAs in this review used the adenoma detection rate (ADR) as the measure of efficacy, not the mean number of adenomas per person (APP), Because of the increasing quality of colonoscopy, the latter has been advocated as a more appropriate measure of efficacy in CPA trials.^29,30^ An agent might reduce the APP but not the ADR. For example, in the seAFOod trial, aspirin did not show an effect on ADR. However, aspirin was associated with a 22% reduction in the APP.^29^

The preventive effects of CPAs often differ by patient subgroups, and chemoprevention might be more effective if targeted to the most appropriate population. For example, a body of literature has shown that aspirin works differentially among patient subgroups. In a RCT with low-dose aspirin among Japanese patients, a qualitative effect modification by smoking was observed: former and never smokers had marked reduction in CRA (OR: 0.37, 95% CI: 0.21-0.68), whereas current smokers had increased risk with aspirin use (OR: 3.45, 95% CI: 1.12-10.64).^31^ A cross-sectional study among the US population observed similar effects, that aspirin only protected against colorectal polyps among nonsmokers and this effect was lost among current smokers.^32^

CPAs may also have differential efficacy against different subtypes of CRC and CRA. CRC is a heterogeneous disease with various histological features, driver mutations, and molecular characterization.^33^ For example, in the seAFOod trial, aspirin was shown to reduce the number of adenomas in the right colon, particularly serrated adenomas, but was also associated with reduced risk of conventional colorectal adenomas. In contrast, eicosapentaenoic acid had an effect on left-sided and conventional colorectal adenomas but not on right-sided or serrated lesions.^29^ To date, most trials have not captured detailed information on the subtype of cancer or adenoma being prevented. Consequently, subtype and location-specific effects may have been missed and the benefits of these agents might have been underestimated.

Genetic variation is another important biomarker that moderates the effect of CPA. Several single nucleotide polymorphisms (SNPs) have been identified to modify the effect of aspirin on CRA or CRC prevention.^34-42^ For example, a genotype analysis of the seAFOod Polyp Prevention Trial data showed that the chemoprevention effect by aspirin was modified by the SNPs in cyclooxygenase, lipoxygenase, and TP53 Genes.^42^ Participants with certain SNP genotypes of these genes benefited more from aspirin use than participants with other alleles. Likewise, although no NMAs found a protective effect of vitamin D supplementation, people with certain genetic traits might still benefit from it. One example is the genotype of vitamin D receptor. A RCT discovered that the effect of vitamin D supplementation is moderated by the rs7968585 allele.^43^ The AA genotype reduced risk by 64%, while AG or GG genotype increased risk by 41%. Recently, a secondary analysis of a vitamin D/calcium intervention RCT showed that individuals with certain vitamin D-binding protein isoforms may particularly benefit from vitamin D supplementation for CRA prevention.^44^ With the advancement of precision prevention, it is very likely that chemoprevention of CRA/CRC can be personalized.^45,46^

Meanwhile, the search for repurposed CPAs is ongoing. It is challenging to find a good CPA. Due to the long duration of use, the CPA must be effective, have minimal side effects, and ideally, low cost. A few CPAs have received attention and are the subject of ongoing research, including metformin, berberine, and more recently, glucagon-like peptide-1 receptor agonists (GLP-1RA).

Metformin is a biguanide compound often used to treat type 2 diabetes. It possesses anticancer properties through a number of mechanisms including the activation of AMP-activated protein kinase, which inhibits the mTOR pathway that promotes cell proliferation.^47^ Observational studies show that metformin reduces the incidence of advanced CRA and CRC among diabetes patients.^48^ To date, there is only one RCT on metformin and chemoprevention of sporadic CRA that used endoscopy-confirmed CRA rates as outcome.^49^ Risk of CRA was significantly reduced among the metformin group. However, due to a short 1-year follow-up of this trial, the long-term effects of metformin on CRC prevention remains unclear. A recent target trial emulation using electronic health records suggests that metformin does not reduce the 6-year risk of CRC either among diabetes patients or patients regardless of diabetes status, which casts doubts on metformin as a CPA.^50^

Berberine is an alkaloid compound in several plants such as barberry and Chinese goldthread.^51^ It is commonly used in traditional Chinese medicine to treat infections and diarrhea, and as adjuvant treatment of type 2 diabetes, hyperlipidemia, and hypertension.^52^ Preclinical studies suggest that berberine has anti-inflammatory properties and potentially anti-tumor effects by activating AMP-activated protein kinase, one of the same targets as metformin.^53^ Berberine is well-tolerated in general, with mild AEs.^54^ However, we showed that its efficacy is inconsistent between the two NMAs that included this agent. Whether it is a good candidate for the prevention of CRC requires further investigation with RCTs of larger sample size and longer follow-up.

GLP-1RAs are a class of agents which mimic the endogenous hormone GLP-1. They are used to treat type 2 diabetes through the stimulation of insulin secretion.^55^ GLP-1RAs are also effective in weight loss by delaying gastric emptying, and by decreasing appetite and craving through a complex signaling to the brain.^56^ Because of the dual effects of GLP-1RAs, they have been proposed to act as a CPA for CRC. A recent retrospective cohort study showed that drug-naïve diabetic patients treated with GLP-1RAs had 44% reduced risk of CRC (HR: 0.56, 95% CI: 0.44-0.72) compared to propensity-score matched patients treated with insulin.^57^ If this association can be ascertained in RCT among both diabetic patients and people with obesity, GLP-1RAs could be a highly effective CPAs in these individuals who are at elevated risk of CRC.

In this review, we focused on the chemoprevention of CRA and CRC. However, it is equally important to emphasize other prevention strategies. Considerable research has elucidated the impact of healthy lifestyle as an important prevention strategy, such as avoiding smoking, reducing alcohol consumption, having a healthy diet, proper weight management, and adequate physical activity.^58-60^ Furthermore, organized early detection strategies are crucial for reducing CRC-related mortality. Both endoscopy-based tests and stool-based tests have demonstrated great benefits for both average and high-risk populations.^3,61^ Blood tests, such as the cell-free DNA test, have emerged as an alternative screening approach with potentially better uptake and adherence.^62^ Finally, for high-risk populations with family history of CRA/CRC, genetic counseling should be considered to help guide prevention and early detection.^63^

### Limitations

Although all NMAs were synthesized from RCT studies, there are still notable heterogeneities and potential for bias. Because of different research questions, each NMA had somewhat different inclusion and exclusion criteria. For example, some NMAs focused only on high-risk population while some also included average-risk population. Therefore, a different number of RCTs might be included even for the same intervention. Researchers might choose to administer different doses of a CPA (e.g., celecoxib, ASA) as different interventions, or choose to group as one intervention. RCTs also had varying durations of intervention, which might violate the transitivity assumption of NMA. However, most NMAs did not address this concern.

We did not pool the estimates from all NMAs in part because of the heterogeneity. Moreover, there was substantial overlapping in the included RCTs in each NMA. Pooling the estimates violates the independent observation requirement for meta-analysis. We only included NMAs of English language and we did not search grey literature for additional NMAs, although the likelihood of missing high-quality NMAs was very low. Lastly, we did not discuss all interventions from the NMAs. For example, we did not present the findings for balsalazide, metformin, eicosapentaenoic acid, resistant starch, and some combinations of CPA such as ASA and DFMO, ASA and EPA.

## Conclusion

In summary, our review of NMAs on chemoprevention of CRA/CRC suggests that low-dose aspirin is currently the best option, considering the balance of efficacy and safety. Nevertheless, knowledge gaps remain on important aspects such as the characteristic of patients associated with better prevention efficacy, the personalized timing and dosing of intervention, new targets for prevention, and the coordination between chemoprevention and surveillance to achieve optimal real-world effectiveness. Future research is needed to better characterize the patient subgroups that benefit more from the existing CPAs, to develop a risk prediction tool that is tailored to personalize the dosing and surveillance interval, and to explore new CPAs with improved efficacy that can be used alone or in combination to provide broad and effective prevention of all subtypes of CRC.

## Supplemental Material

Supplemental Material - Review of Network Meta-Analyses on the Efficacy of Chemopreventive Agents on Colorectal Adenomas and Cancer Supplemental Material Review of Network Meta-Analyses on the Efficacy of Chemopreventive Agents on Colorectal Adenomas and Cancer by Yibing Ruan, Chantelle Carbonell, Karen Brown, Robert J. Hilsden, and Darren R. Brenner in Cancer Control

## Appendix

### Abbreviations

ADR: adenoma detection rate
AE: Adverse event
APP: adenomas per person
ASA: Acetylsalicylic acid (aspirin)
CI: confidence interval
Cox-2: cyclooxygenase-2
CPA: chemopreventive agent
CRC: colorectal cancer
CRA: colorectal adenoma
CVD: cardiovascular disease
FAP: familial adenomatous polyposis
GI: gastrointestinal
GLP-1RA: glucagon-like peptide-1 receptor agonists
NMA: network meta-analysis
OR: odds ratio
RCT: randomized controlled trials
RR: risk ratio
NSAID: non-steroidal anti-inflammatory drug
UDCA: ursodeoxycholic acid
