# Supplementary material for: Review of Network Meta-Analyses on the Efficacy of Chemopreventive Agents on Colorectal Adenomas and Cancer
Source: Cancer Control. 2025 May 20;32:10732748251344481. doi: 10.1177/10732748251344481 (PMC12093012; doi:10.1177/10732748251344481)
Supplement: Supplemental Material - Review of Network Meta-Analyses on the Efficacy of Chemopreventive Agents on Colorectal Adenomas and Cancer [file sj-pdf-1-ccx-10.1177_10732748251344481.pdf]

Table S1. Search strategy and results of the PubMed database

Step	Search term	Results
#1	"adenoma"[Title/Abstract] OR "adenomatous polyp*"[Title/Abstract]	69,840
#2	colon cancer[Title/Abstract] OR colonic neoplasm*[Title/Abstract] OR colon carcinoma[Title/Abstract] OR colon tumor*[Title/Abstract] OR colon tumour*[Title/Abstract] OR colonic polyp*[Title/Abstract]	77,568
#3	"colorectal cancer"[Title/Abstract] OR "colorectal neoplasm*"[Title/Abstract] OR "colorectal carcinoma"[Title/Abstract] OR "colorectal tumor*"[Title/Abstract] OR "colorectal tumour*"[Title/Abstract] OR "colorectal polyp*"[Title/Abstract]	159,232
#4	rectal cancer[Title/Abstract] OR "rectal neoplasm*"[Title/Abstract] OR "rectal carcinoma"[Title/Abstract] OR "rectal tumor*"[Title/Abstract] OR "rectal tumour*"[Title/Abstract] OR "rectal polyp*"[Title/Abstract]	36,627
#5	colon*[Title] OR rectal[Title] OR rectum[Title] OR colorectal[Title] OR intestin*[Title]	500,326
#6	chemoprevent*[Title/Abstract] OR Chemoprophylaxis[Title/Abstract] OR prevent*[Title/Abstract]	1,908,536
#7	network meta-analysis[Title]	8,871
#8	(#1 OR #2 OR #3 OR #4) AND #5 AND #6 AND #7	14

Table S2. Chemoprevention mechanism of selected CPAs

Agent	Proposed mechanism in CRA/CRC prevention
Celecoxib	Celecoxib is a selective inhibitor of COX-2, an enzyme involved in prostaglandin (PGE2) synthesis. PGE2 promotes cell proliferation, inhibits cancer cell apoptosis, and promotes cancer metastasis through multiple signaling pathways. Celecoxib also promotes cancer cell death through mitochondrial apoptosis mechanism.
Sulindac	Sulindac is a non-selective NSAID which targets COX enzymes (COX-1 and COX-2). Sulindac can also inhibit cell growth and induce apoptosis through inhibiting cyclic guanosine monophosphate phosphodiesterase (cGMP PDE) activity, leading to increased intracellular cGMP levels. This

	in turn activates cGMP-dependent protein kinase (PKG), which can suppress beta-catenin pathway and downregulate cyclin D1 and survivin.
Aspirin	Aspirin can directly inhibit the activity of COX-2, therefore inhibiting cancer cell proliferation and promoting apoptosis. Alternatively, it was proposed that ASA inhibits COX-1 in platelets and reduces platelet activation, a hypothesized important step of colorectum carcinogenesis. Platelet activation results in local recruitment of immune cells, leading to inflammation and indirect increase in COX-2 activity in colorectal tissues.
Difluoromethylornithine (DFMO)	DFMO blocks polyamine synthesis through the irreversible inhibition of ornithine decarboxylase (ODC). Polyamines are essential for rapid cell proliferation and tumor growth. Suppressing the production of polyamines could slow down the process of carcinogenesis.
Calcium	The hypothesized mechanism of calcium in inhibiting colorectal carcinogenesis is binding to fatty acids and bile acids in the colon to reduce their inflammation-promoting effects on colon epithelial cells. Additionally, calcium could also modulate immunity by regulating T-cell proliferation and differentiation, and reduce tumour-inflammation markers.
Vitamin D	Vitamin D exerts anti-proliferative effects in colon cells through inducing cell cycle arrest through upregulating p21WAF1/CIP and p27KIP and inducing apoptosis via the mitochondria pathway. Vitamin D also exerts anti-inflammatory effect by interfering with the synthesis of prostaglandins, stress-activated kinase signaling, and production of proinflammatory cytokines.
Folate/folic acid	Folate is an essential cofactor for the de novo biosynthesis of purines and thymidylate, therefore playing an important role in DNA synthesis and replication. Folate deficiency increases the risk of neoplastic transformation of colon cells with aberrant DNA methylation patterns and impaired DNA stability. However, folate possesses dual modulatory effects on colorectal carcinogenesis depending on the dose of folate intervention. Modest supplemental levels (4–10 times above the basal dietary requirement) suppress whereas supraphysiological supplemental doses enhance the development of CRC.
Antioxidant	Oxidative stress (OS), an imbalance between reactive oxygen species (ROS) and antioxidant defenses, plays a significant role in colorectal carcinogenesis. OS damages DNA, causing mutations that can promote uncontrolled cell growth and cancer progression. OS can also affect other cellular processes, including inflammation and immune evasion, further contributing to the development and progression of cancer. Therefore, antioxidants from foods and supplements such as flavonoids are proposed to protect against cancer development.

Berberine	Berberine is an alkaloid compound commonly used in traditional Chinese medicine to treat infections and diarrhea, and as adjuvant treatment of type 2 diabetes, hyperlipidemia, and hypertension. Berberine may possess anti-inflammatory properties and potentially anti-tumor effects by activating AMP-activated protein kinase (AMPK), which can suppress cancer cell proliferation through negatively regulating the mTOR pathway.
Ursodeoxycholic acid (UDCA)	UDCA is a synthetic bile acid. The hypothesized mechanism of benefit includes an immunomodulatory effect, a direct cytoprotective effect, a choleretic effect by increasing bile flow, and an indirect cytoprotective effect by displacement of hydrophobic bile acids.
